# Occupational Allergic Contact Dermatitis to Methylisothiazolinone in an Engobe Ceramic Decorator

**DOI:** 10.1111/cod.70126

**Published:** 2026-03-19

**Authors:** Paolo Antonio Danza, Giulia Ciccarese, Françoise Giordano‐Labadie, Teresa Nocera, Caterina Foti

**Affiliations:** ^1^ Interdisciplinary Department of Medicine, Section of Occupational Medicine University “Aldo Moro” of Bari Bari Italy; ^2^ Unit of Dermatology, Department of Medical and Surgical Sciences University of Foggia Foggia Italy; ^3^ Dermatology Department Paul Sabatier‐Toulouse III University Toulouse France; ^4^ Department of Precision and Regenerative Medicine and Jonian Area Section of Dermatology, University “Aldo Moro” of Bari Bari Italy

**Keywords:** allergic contact dermatitis, engobe ceramic decorator, methylisothiazolinone, occupational dermatitis, patch test

Methylisothiazolinone (MI) is a synthetic antimicrobial often employed in personal care products [1, 2]. The last European Union regulation governing the use of MI in cosmetics, which took effect in 2017, permitted this ingredient only in rinse‐off cosmetic products at a maximum concentration of 0.0015% (15 ppm) [3]. However, the use of MI in industrial products is not restricted and cases of occupational ACD (OACD) associated with MI have been described [4, 5].

## 1 Case Report

A 34‐year‐old woman presented to the Occupational Diseases Ward at the University Hospital of Toulouse, France, with a history of hand dermatitis that had been present for approximately 3 years. In the last year, the cutaneous lesions had become larger and itchier. The patient's medical history included only atopic dermatitis (AD).

**FIGURE 1:**
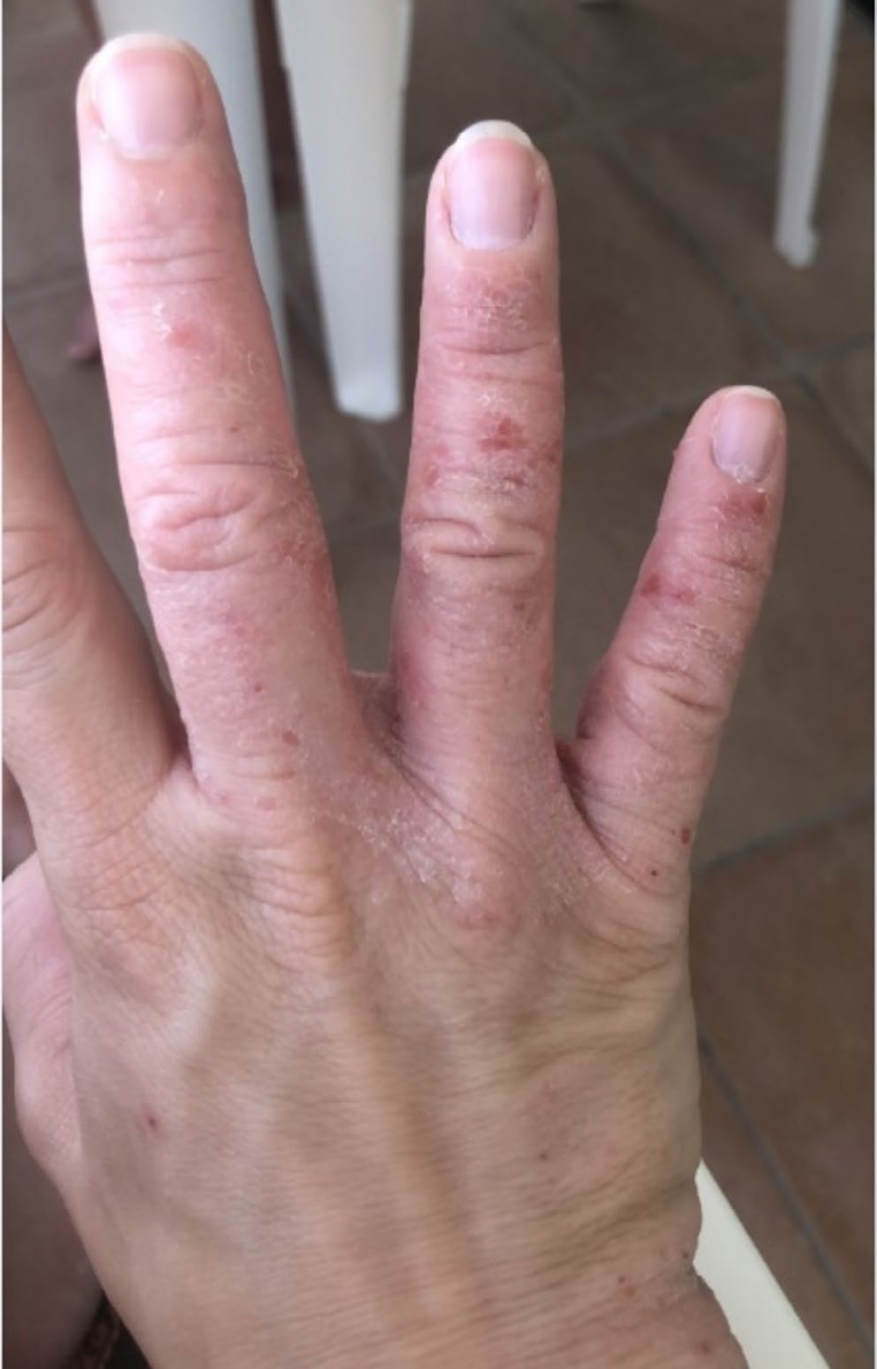
Eczematous dermatitis on the back of the hand and fingers with erythema, scaling, and scattered excoriations.

On clinical examination, erythematous papules and plaques were observed on both hands, including the palms and backs, sometimes covered by vesicles and bleeding fissures (Figure 1). The patient did not report any changes in her lifestyle or personal care products. She has been working for 5 years as an engobe ceramic decorator at a company that manufactures earthenware products. Her work involved applying different colorways to the tiles using water‐based paints. She claimed to wear at work the personal protective equipment (PPE) provided by her employer, such as short‐sleeved nitrile gloves, goggles, smocks, aprons and safety footwear. The patient applied a corticosteroid cream (desonide 0.1%) to her hands, which resulted in only partial improvement. After a workplace accident, she was absent from work for six months. Following a 30‐day period, she experienced a complete spontaneous recovery. Notably, during her sick leave, she was visited, and no skin lesions were detected.

**FIGURE 2:**
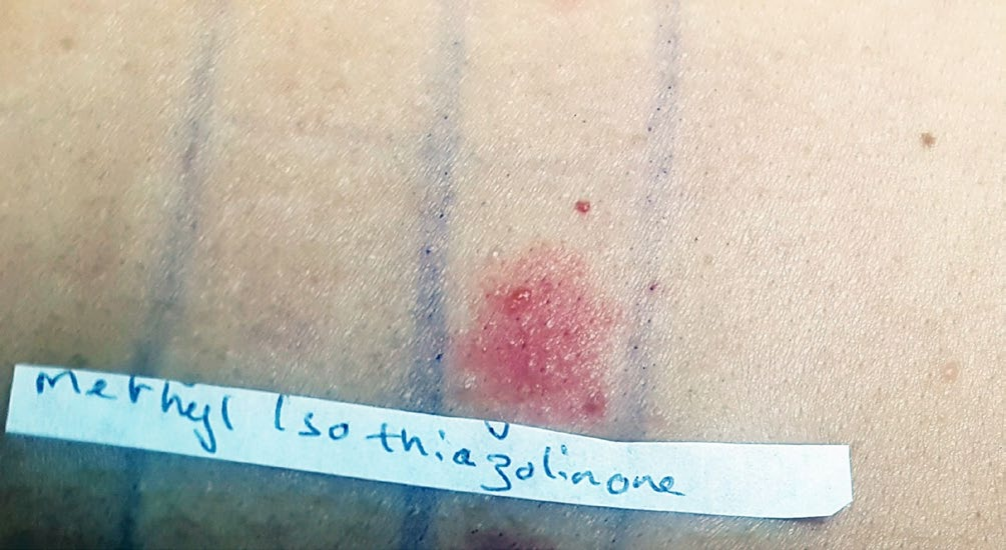
Day‐3 patch test reaction to methylisothiazolinone (MI) 0.2% aq showing a ++ positive response.

An OACD was suspected. Patch tests were performed using the European Baseline Series and the French additional series of the vigilance network in Dermato‐Allergology, using IQ‐Ultimate patch test chambers (Laboratoire Destaing, Grasse, France) on Oper tape (Herbitas Laboratorios, Foios‐Valencia, Spain) [6, 7]. The occlusion time was 2 days, and results were read on days 2 and 3 according to the criteria of the European Society of Contact Dermatitis, revealing a strong positive reaction to MI 0.2% aqueous (++) (Figure 2) [6, 7]. Personal hygiene products (specific for atopic skin) and work gloves have been equally tested and were negative. The 7‐day reading was taken using a photo, which showed persistence of MI positivity with no further positive reactions [6, 7]. Notably, MI was listed as an ingredient in the safety data sheet of the paint used by our patient. The eczematous reaction definitively resolved after avoiding exposure to any MI‐containing products at work (the patient used only MI‐free paints thereafter) and by using long‐sleeve (instead of short‐sleeve) gloves in addition to other PPE. The patient was also instructed to check for MI content in all rinse‐off cosmetics and personal care products and to avoid them. At the three‐month follow‐up visit after treatment and upon her return to work, no relapse of ACD was observed. The patient gave written informed consent to publish this case.

## 2 Discussion

Our patient developed an ACD due to contact with water‐based paint containing MI. The relevance of the occupational setting is supported by several factors: the temporal association between the patient's exposure to the water‐based paint and the clinical manifestation, the complete recovery when she was not exposed to MI, the patch test positive reaction to MI and the fact that the safety data sheet of the paint used by our patient listed MI as one of the ingredients. Moreover, the patient's history of AD represented a predisposing factor to ACD [8].

MI‐induced ACD has increased since its widespread deployment [4, 9], with most cases reported in occupational settings, including healthcare and beauty workers, hairdressers, and metal workers [2, 5, 10]. Among manufacturing workers, those handling water‐based paints and varnishes are at risk of developing OACD, with a high ACD share on the face, because they are exposed to a variety of contact allergens and skin irritants in paints and varnishes, adhesives, preservatives, solvents and detergents, fillers (putty), or nitrile rubber gloves [10, 11]. However, an OACD of the hand induced by MI in a painter who works as an engobe ceramic decorator, as our patient, has never been described so far.

We supposed our patient was exposed to the paint, and therefore to MI, mainly through skin contact: the use of short gloves might not have completely covered the hands and might have allowed some contact, even if minimal, with the product being handled.

Although patch testing with nitrile gloves “as is” was negative in our patient, a role of the gloves cannot be completely excluded. A recent Finnish study demonstrated that disposable nitrile rubber gloves may contain isothiazolinone preservatives, including methylisothiazolinone; therefore gloves may represent a potential hidden source of exposure in sensitised individuals [12]. In this context, a Glove Repeated Application Test (GRAT) may be considered to further assess a possible glove‐related contact dermatitis; although additional data are needed to support its routine application [13].

The revised Classification, Labelling and Packaging of chemicals (CLP) Regulation of the European Union [14], which entered into force on 10 December 2024, enhanced information transparency, imposing that labelling will be made simpler and that it has to contain information on chemical hazards. Indeed, the fact that MI was listed in the safety data sheet of the paint used by our patient enabled us to solve the case.

Regretfully, to date, no restriction on the use of MI has been applied to products not listed as cosmetics or personal care goods.

As our case demonstrates, professional categories using water‐based paints, such as ceramic decorators, are at risk of developing ACD to MI, particularly in the presence of predisposing factors, such as a compromised skin barrier in AD patients.

Regulatory measures on the use of MI should cover not only cosmetics but also products related to work activities in the chemical, industrial, and artisan sectors.

## Author Contributions

**Paolo Antonio Danza:** conceptualization, investigation, writing – original draft, formal analysis, data curation. **Giulia Ciccarese:** conceptualization, writing – review and editing, supervision. **Caterina Foti:** supervision, data curation, visualization. **Françoise Giordano‐Labadie:** contributed to investigation and validation of our manuscript. **Teresa Nocera:** contributed to Investigation and Validation of our manuscript.

## Funding

The authors have nothing to report.

## Conflicts of Interest

The authors declare no conflicts of interest.

## Supporting information


**Data S1:** Supporting Information

## Data Availability

The data that support the findings of this study are available from the corresponding author upon reasonable request.
