# Supplementary material for: Occupational Allergic Contact Dermatitis to Methylisothiazolinone in an Engobe Ceramic Decorator
Source: Contact Dermatitis. 2026 Mar 19;94(6):694–6. doi: 10.1111/cod.70126 (PMC13139699; doi:10.1111/cod.70126)
Supplement: Supplementary file 1 — Data S1: Supporting Information [file COD-94-694-s001.pdf]

Checklist for Submission of Case Reports as "Contact Points"[1]

	**Reporting should include**	page
1	Full title page compliant with general author guidelines of the journal	1
2	Appropriate key words, including "case report"	1
3	CAS no., INCI or INN nomenclature, where applicable used throughout text, tables and figure legends	2
4	Introduction of normally two to three sentences	2
5	Headings: Case Report & Discussion at appropriate points	2
6	Basic patient information: age, sex	2
7	Additional patient information as applicable: occupation, leisure activities	2
8	Patient's history and symptoms	2
9	Medical history, focusing on relevant exposures	2
10	Clinical findings: Precise anatomical site, morphology, …	2
11	Only if useful, tabular timeline, e.g., in cases with multiple exposures causing CADR	-
12	Patch test technique, detailing brand / manufacturer of chambers and adhesive tape, supplier of hapten preparation, adherence to international standards (e.g., ICDRG/ESCD[2]), **exposure time used, reading times used, concentration and vehicle of haptens** (including patient's materials) should be documented. Modifications of standard technique mentioned.	2, 3
13	Other tests (open or semi-open test, ROAT, PUT, intradermal) described and results reported	-
14	In case of newly described haptens, control tests (ideally in n=20) performed with preparation and outcome reported.	-
15	Clinical relevance examined, basis (product information, chemical analysis, …) and result reported.	3
16	In case of immediate type hypersensitivity testing, relevant tests with controls and possibly laboratory results	-
17	Discussions of strengths and limitations (difficulties encountered)	3
18	Relevant medical literature (usually up to 10 references)	3
19	Supplemental details accommodated separately in "Online Supplemental Material" (The Editor may also advise on this after review)	-
20	A main conclusion for the clinician, or concerning research given at the end highlighting how the paper adds to existing knowledge	3
21	Patient's written consent obtained and submitted if images are provided which show him or her in a recognisable fashion. The same is recommended if the description is so detailed that the patient's profile may be recognised from the information given, following national / institutional standards	-

[1] Based on: Uter W, Goossens A, Gonçalo M, Johansen JD. Guidelines for the presentation of contact allergy case reports. Contact Dermatitis 2017:76:107-113 (http://onlinelibrary.wiley.com/doi/10.1111/cod.12709/abstract)

[2] Johansen JD, Aalto-Korte K, Agner T et al. European Society of Contact Dermatitis guideline for diagnostic patch testing – recommendations on best practice. Contact Dermatitis 2015:73:195-221 (http://onlinelibrary.wiley.com/doi/10.1111/cod.12432/abstract)
